# Development and application of the vaccine reporting quality score to evaluate the vaccine adverse event reporting system

**DOI:** 10.3389/fimmu.2026.1871141

**Published:** 2026-08-06

**Authors:** Zongkai Zhou, Zirong Mu, Nina Gu, Xu Sun, Zun Wu, Zuguo Tian, Wanhong Luo

**Affiliations:** School of Physical Education, Hunan University, Changsha, Hunan, China

**Keywords:** data quality, evaluation tool, pharmacovigilance, spontaneous reporting, vaccine adverse event reporting system, vaccine reporting quality score, vaccine safety

## Abstract

**Background and aims:**

Ensuring the documentation quality of spontaneous reports in the Vaccine Adverse Event Reporting System (VAERS) is important for signal detection, yet targeted tools for evaluating vaccine-specific reporting quality remain lacking. This study proposes the Vaccine Reporting Quality Score (VRQS)—a transparent, exploratory evaluation system—and applies it to a large-scale VAERS dataset.

**Methods:**

The preliminary VRQS aggregates 13 items into two primary domains: Completeness (items 1-10) and Clinical Informativeness (items 11-13), using equal weighting as an initial exploratory index. Using the VAERS 2025 dataset, we established a strict 1-to-1 patient case unit of analysis (N = 40,285 unique case reports) to evaluate demographic, administrative, and clinical density. Multivariable linear and ordinal logistic regressions were executed using independent case characteristics, with all overlapping score components removed *a priori* to eliminate structural circularity.

**Results:**

The mean overall quality score was 9.04 ± 1.96, with 57.54% of unique reports falling into a medium-quality tier (scores 8–10). Product identifiers, such as vaccine type (97.92%), were highly prevalent, whereas informative laboratory data were present in only 16.18% of records. Distinct, descriptive documentation profiles emerged: spontaneous records displayed high completeness but low informativeness, whereas professional entries and serious adverse events (SAEs) featured dense clinical metrics alongside missing administrative checkfields. In re-specified, non-circular multivariable models, complex clinical factors such as pre-existing allergies (b=0.832, p<0.001) and concurrent medications (b=0.463, p<0.001) were independently associated with higher quality score.

**Conclusion:**

The proposed VRQS framework demonstrates that administrative documentation density does not inherently correlate with clinical informativeness. Rather than acting as a validated tool for causality assessment, the VRQS provides a transparent, preliminary approach for report prioritization and screening pending formal external validation against global benchmarks.

## 1 Introduction

The robustness of post-marketing vaccine safety surveillance depends fundamentally on the quality of Individual Case Safety Reports (ICSRs) submitted to spontaneous reporting systems (1). The Vaccine Adverse Event Reporting System (VAERS) serves as the primary national cornerstone for vaccine safety in the United States (2). Established in 1990 and co-managed by the Centers for Disease Control and Prevention (CDC) and the Food and Drug Administration (FDA), this infrastructure acts as a centralized repository designed to provide real-time surveillance of potential health concerns related to immunization programs (3). Beyond its foundational role, it functions as the country’s largest reporting system for monitoring adverse events following immunization (AEFIs) (4). As a national early-warning system, it provides a vital platform for detecting potential safety signals that may not have been observed during clinical trials (2).

However, as a passive surveillance system, VAERS is inherently subject to several critical limitations, most notably underreporting and variable data quality (5). Reports are submitted voluntarily by a wide range of individuals, including healthcare professionals, manufacturers, and the public (2). The data often suffers from inconsistent documentation, missing clinical details, and a lack of verified medical evidence. For instance, a recent review of the 2025 VAERS dataset revealed significant documentation gaps, identifying that one-fifth of the reports lacked patient age and nearly one-third omitted administration site information (6). These limitations underscore the urgent need for a systematic framework to evaluate the quality and reliability of the information in these reports, ensuring they can effectively guide public health decisions.

Crucially, researchers and software developers must maintain a strict conceptual boundary between a report’s data completeness (the clerical presence of expected fields), its clinical informativeness (the density of qualitative and objective medical information), and its clinical credibility or causal validity (the verifiable truth of the event or the probability that the vaccine biologically caused the injury) (7). A reporting quality score cannot verify clinical truth or confirm biological causality. An entry containing an extensive, grammatically thorough narrative may be entirely speculative or clinically uninformative, whereas a brief clinician note may convey a decisive, high-value medical truth. Spontaneous safety monitoring indices assess documentation density and structural consistency; they do not serve as arbiters of biological fact.

In the broader landscape of pharmacovigilance, the importance of evaluating case quality has been recognized through the development of tools such as VigiGrade (8). It was introduced by the Uppsala Monitoring Center to identify well-documented ICSRs within the WHO global database (8). VigiGrade establishes a valuable benchmark by applying an initial score of 1.0 and deducting non-linear, fractional penalties for missing details across 10 structured dimensions (9). However, VigiGrade relies on complex algorithmic weights that introduce significant computational overhead, creating operational barriers for routine, real-time database filtering or rapid clinical screening by frontline research staff. Furthermore, VigiGrade is optimized for generalized drug-exposure reporting rather than the specific administrative architecture, regulatory checkfields, and immunization parameters intrinsic to the U.S. VAERS network. Crucially, VigiGrade completely lacks tracking parameters for product traceability fields critical to vaccine manufacturing and safety actions, such as vaccine type, manufacturer, and lot/batch numbers.

Despite the critical role VAERS plays in public health, a significant research gap remains: the lack of a standardized, comprehensive, and easy-to-use evaluation system specifically designed for vaccine-related reports. Existing assessment methods often rely on manual, labor-intensive reviews of individual narratives or focus strictly on a narrow set of mandatory fields, which fail to capture the multidimensional nature of reporting quality in a high-volume data environment (10). This absence of a tailored metric makes it difficult for researchers and regulators to systematically quantify reporting trends or identify specific factors that drive high-quality documentation.

To address this gap, the current study introduces the Vaccine Reporting Quality Score (VRQS) as an exploratory, simple, and transparent index designed for large-scale database screening and report prioritization. The primary intended users of this tool are pharmacovigilance researchers, epidemiologists, and regulatory analysts who require scalable mechanisms to group large datasets or execute parallel sensitivity analyses across varying documentation tiers before deploying signal-detection algorithms. VRQS is not intended for frontline reporters, for clinical decision-making, or for causal inference, and is presented throughout as an exploratory framework pending external validation. By mapping 13 core items into two distinct descriptive tracks—Completeness and Clinical Informativeness—this proposed, unvalidated tool is applied to a complete-case cohort from the VAERS 2025 database to characterize baseline documentation patterns and identify independent clinical correlates of reporting quality.

## 2 Methods

### 2.1 Data source and unit of analysis

We conducted a cross-sectional data-processing analysis using the publicly available VAERS 2025 dataset. The raw VAERS architecture is structurally distributed across three distinct, relational data tables linkable by a unique VAERS identification number (VAERS_ID): the Demographics file, the Symptoms file, and the Vaccine file (11). This study utilized de-identified, publicly available data and was therefore deemed exempt from ethical review by the Institutional Review Board of Hunan University. Informed consent was also not required because the analysis was retrospective and used de-identified data. The research was conducted in accordance with the tenets of the Declaration of Helsinki and applicable federal laws regarding the use of public surveillance data.

To prevent statistical distortion and the artificial inflation of standard errors and *p*-values caused by one-to-many join duplications during table merges, the analytic unit for this study was strictly defined as the unique individual VAERS report (one row per unique VAERS_ID). Starting with 40,804 crude demographic entries, we systematically removed 519 duplicate or updating administrative IDs, yielding exactly 40,285 unique patient cases. For the 25.75% of reports that documented multiple concurrent vaccinations, the underlying vaccine-specific rows were collapsed into a single report-level array, where vaccine-level items (such as vaccine type, manufacturer, lot number, and dose series) were scored as complete if the required data were present for at least one of the administered vaccines. This data-cleaning pipeline established a final analytical cohort of exactly 40,285 unique case reports, ensuring a fixed, unduplicated denominator for all subsequent data-density metrics, statistical tests, and regression parameters.

### 2.2 Data collection

Data fields were extracted from the linked VAERS tables and mapped into five operational dimensions:

#### 2.2.1 Demographics

Patient age was treated as a continuous variable and stratified into pediatric (<18 years), adult (18–64 years), and elderly (≥65 years). Biological sex was recoded into male and female. Geographic data from individual U.S. states and territories were aggregated into five distinct U.S. Census Bureau regions: Northeast, Midwest, South, West, and territories.

#### 2.2.2 Clinical history

We documented the history of known allergies and prior adverse reactions to vaccinations. The clinical state at the time of vaccination or reporting was captured through current illness (presence of acute illness), medical history (underlying chronic medical conditions), and medication use (concurrent medications). These variables were coded as binary indicators (yes/no) to identify pre-existing conditions or clinical context. Additionally, symptom information was extracted from the original free-text narratives in the dataset.

#### 2.2.3 Administration

Vaccine administration site was reclassified into public (including government-funded or community-based settings), private (including private healthcare providers and clinics), and other. The reporter type was categorized by the report source as either spontaneous (consumer/patient) or professional (healthcare provider/manufacturer).

#### 2.2.4 Vaccine information

Four vaccine-related variables were collected: specific vaccine type (e.g., COVID-19, shingles, and flu), manufacturer (e.g., Glaxosmithkline Biologicals, Merck, and Moderna), lot/batch number, and dose series (e.g., first, second, and third).

#### 2.2.5 Case outcomes

Serious adverse event (SAE) was coded as a binary variable (yes/no) based on the presence of any of the following criteria: death, life-threatening illness, permanent disability, inpatient hospitalization (or prolongation of existing hospitalization), or congenital anomalies (12). Recovery status was classified as yes (recovered), no (not recovered at the time of reporting), and unknown. Deaths are captured within the SAE composite (death is one of the five criteria) and are not analyzed as a separate subgroup; the small number of death reports precludes stable subgroup analysis. At the same time, their recovery status was cross-checked and classified under the “No” (unrecovered) domain to preserve the clinical trajectory within the data density matrix.

All missing fields, blank values, and invalid entries were explicitly classified into the “Unknown” (UNK) category to maintain sample integrity and prevent selection bias due to listwise deletion.

### 2.3 Construction of the vaccine reporting quality score

To systematically map the documentation density of passive surveillance records, we developed the Vaccine Reporting Quality Score (VRQS), a 13-item exploratory index divided into two primary domains. Domain I (Completeness; Items 1–10) assesses the presence of core epidemiological, administrative, and product-traceability fields necessary for basic case tracking. Domain II (Clinical Informativeness; Items 11–13) captures descriptive narrative depth, clinical source tracking, and objective diagnostic text fields. We map each VigiGrade dimension to one of three fates in VRQS (**Supplementary Table 1**): (a) retained (e.g., age, sex, time-to-onset); (b) modified (e.g., reporter type recoded to the VAERS CATEGORY field; free-text length threshold re-tuned for VAERS narrative conventions); or (c) dropped (e.g., indication, because VAERS does not include a structured indication field at the report level). VRQS additionally retains two vaccine-specific traceability items (lot number and dose series) that VigiGrade, designed for general drug ICSRs, does not emphasize.

To maximize transparency and ease of computation for large-scale data-screening operations, the VRQS applies an equal-weighting scheme in which each item is scored as 1 if the specific criteria are satisfied and 0 otherwise, yielding a cumulative score range of 0 to 13. This additive weighting framework serves as a simple, exploratory index rather than a finalized psychometric measurement instrument; it provides a transparent baseline that avoids the subjective or data-dependent biases inherent in complex algorithmic weighting models. Consequently, the tool did not undergo formal Receiver Operating Characteristic (ROC) analysis or expert consensus Delphi rounds, and its cut points are proposed purely as descriptive groupings.

To ensure high specificity and protect the index from distortion by superficial data entries, a stringent placeholder-filtering protocol was implemented for all evaluated variables. All raw string fields were systematically converted to uppercase, trimmed of trailing and leading whitespace, and evaluated against a regular expression matrix specifically designed to isolate non-informative text placeholders such as “UNK”, “UNKNOWN”, “NONE”, “NA”, “N/A”, “NOT PROVIDED”, “.”, and “?”. If a field contained only placeholder tokens, it was stripped of its validity and scored as 0. This automated data-cleaning filter ensures that the resulting score reflects genuine data density rather than clerical boilerplate text entry.

### 2.4 Operational definitions

**Table 1** and **Supplementary Material S1** provide complete operational definitions and mapping codes for all 13 items. Key rules:

**Table 1:** Principles, operational definitions, and overview of the vaccine reporting quality score (VRQS) system.

Domain/ item	Data dimension	Variable	Principle	Operational definition (detailed codebook in Supplementary Material S1)
Completeness domain (VRQS 1-10)				
VRQS1	Demographics	*AGE_YRS*	Age stratification for age-specific safety signals.	Non-missing and not equal to a placeholder token. If AGE_YRS is missing, CAGE_YR (years, computed by VAERS) and CAGE_MO (months) are consulted in order.
VRQS2	Demographics	*SEX*	Sex-based analysis for biological differences in reporting.	Non-missing AND not equal to a placeholder AND not equal to “U”. Valid informative values: F, M.
VRQS3	Geography	*STATE*	Spatial surveillance for regional cluster detection.	Non-missing and not equal to a placeholder. Valid values: 2-letter US state codes and US territory codes.
VRQS4	Temporal	*ONSET_DATE; VAX_DATE; RECVDATE*	Chronological consistency of exposure, event, and report dates.	0 <= (ONSET - VAX) <= 365 d AND 0 <= (RECV - ONSET) <= 365 d. Same-day scored 1; missing or out-of-range scored 0.
VRQS5	Timeliness	*RECVDATE; VAX_DATE*	Administrative timeliness; supports early-signal detection.	RECV non-missing AND VAX non-missing AND RECV >= VAX.
VRQS6	Administrative	*VAX_ADMIN_BY*	Institutional traceability; contextualizes reporting standards.	Non-missing and not a placeholder; VAX_ADMIN_BY code 9 (Unknown) scored 0, all others scored 1.
VRQS7	Product (type)	*VAX_TYPE*	Platform specificity (e.g., mRNA vs. viral vector).	Non-missing and not a placeholder. Multi-vaccine: report = 1 if satisfied for >=1 vaccine.
VRQS8	Product (manufacturer)	*VAX_MANU*	Manufacturer ID for brand-specific post-market monitoring.	Non-missing and not a placeholder. Multi-vaccine: report = 1 if satisfied for >=1 vaccine.
VRQS9	Traceability (lot)	*VAX_LOT*	Lot/batch number for identifying manufacturing defects.	Non-missing and not a placeholder. Multi-vaccine: report = 1 if satisfied for >=1 vaccine.
VRQS10	Traceability (dose)	*VAX_DOSE_SERIES*	Dose-response (prime, secondary, booster).	Non-missing and not a placeholder. Multi-vaccine: report = 1 if satisfied for >=1 vaccine.
Clinical informativeness domain (VRQS 11-13)				
VRQS11	Source	*SPLTTYPE*	Reports from manufacturer or immunization project (higher data-collection standard).	SPLTTYPE non-missing (manufacturer or immunization project).
VRQS12	Evidence (lab)	*LAB_DATA*	Informative laboratory information (non-placeholder).	Non-missing AND trimmed non-empty AND does not match the garbage regex (none/no/n/a/na/unk/unknown/./-/)?.
VRQS13	Narrative	*SYMPTOM_TEXT*	Narrative length as a coarse proxy for case detail.	strlen(SYMPTOM_TEXT) > 250. Sensitivity at 100/500/1000 chars (Spearman 0.97-0.99; **Supplementary Table 4**).

All items are scored 1 (informative/plausible) or 0 (missing/implausible). Placeholder tokens for the vaccine-level string fields: UNKNOWN, UNK, NONE, NA, N/A, NOT PROVIDED, “.”, and “?”, applied case-insensitively after upper-casing and trimming. Completeness = sum of items 1-10 (range 0-10); Clinical Informativeness = sum of items 11-13 (range 0-3); Total VRQS = sum of items 1-13 (range 0-13). Detailed codebook in **Supplementary Material S1**.

Placeholders excluded. Fields containing only the tokens UNK, U, Unknown, N/A, “None reported,” or other VAERS-native placeholder strings are treated as missing for item scoring.

Multi-vaccine aggregation. For items whose content is vaccine-specific (items 6–9 and 11), the report is scored 1 if the criterion is satisfied for at least one vaccine in the report, and 0 otherwise. The aggregation rule is reported in **Supplementary Table 3** and is implemented in the analysis code.

Plausible onset interval (item 4). Defined as 0 ≤ ONSET_DAYS ≤ 365, where ONSET_DAYS = (date of onset − date of vaccination). Negative values, values > 365 days, and records with missing vaccination or onset dates all receive a score of 0. Same-day onset is scored 1.

Timeliness (item 5). Defined as: the report date is non-missing AND the report date is on or after the vaccination date. Item 13 is intended as a coarse administrative-timeliness marker and does not replace dedicated time-to-onset analyses (item 4) or time-to-report analyses (not currently in VAERS).

Informative laboratory data (item 12). Defined as: LAB_DATA field is non-missing AND non-placeholder.

Clinical narrative length (item 13). Defined as the length of the SYMPTOM_TEXT field in characters. The primary threshold is >250 characters. Sensitivity analyses at 100, 250, 500, and 1,000 characters are reported in **Supplementary Table 4**; rank-order correlation across thresholds is 0.95–0.96.

### 2.5 Statistical analysis

Statistical analyses were performed using Stata/BE 17.0, with significance set at p < 0.05. Means and standard deviations were calculated for the VRQS total and domain scores. We also calculated the frequencies and percentages of satisfied criteria for each of the 13 individual VRQS items. To examine unadjusted differences in reporting quality across case characteristics, bivariate analyses were conducted. Independent-samples t-tests were used to compare mean total VRQS scores and individual domain scores between binary groups, while one-way analysis of variance (ANOVA) was used to compare mean quality scores across multi-level categorical variables. To quantify the true magnitude of these binary disparities independent of the study’s large sample size, Cohen’s *d* effect sizes along with their corresponding 95% confidence intervals (CIs) were systematically calculated. Effect sizes for the bivariate comparisons are reported as Cohen’s d for one-vs-reference contrasts (reference categories shown as the comparison group), with the corresponding 95% CI on d and a Welch’s t-test p-value.

Three multivariable regression models were constructed to identify factors independently associated with the VRQS and the two subdomains. All models adjust for a pre-specified set of demographic, clinical-history, and source characteristics selected *a priori* from the three domains described in §2.2. To prevent mathematical circularity, all individual component items that directly contribute to the calculated score metrics were excluded from the independent variable matrix (e.g., “unknown age” is not used as a predictor in the Completeness model because age presence is item 1). Model 1 used Ordinary Least Squares (OLS) linear regression to examine the impact of clinical histories, case outcomes, and administrative tracking characteristics on the overall VRQS score. Model 2 similarly employed an OLS linear regression model to evaluate the determinants of completeness domain score. Unstandardized coefficients (b) were reported to provide a direct interpretation of the effect size on the scoring scale. Finally, because the clinical informativeness domain yields a tightly bounded range of ordered integers from 0 to 3, an ordinal logistic regression model (ologit) was applied to estimate proportional Odds Ratios (ORs) and their 95% Confidence Intervals (CIs), thereby preserving statistical validity over standard linear assumptions. No predictor exceeded the conventional VIF threshold of 5 in any model, indicating that multicollinearity was not a substantive concern for the reported coefficients. All statistical thresholds were interpreted using two-tailed *p*-values with significance designated at the *p* < 0.05, *p* < 0.01, and *p* < 0.001 levels.

## 3 Results

### 3.1 Performance and distribution of the VRQS

A total of 40, 285 adverse event reports were evaluated using the 13-item VRQS framework, and the results are shown in **Table 2**, **Supplementary Material 3**, **Supplementary Figure 1**. The mean overall quality score was 9.04 ± 1.96 (median = 9.00), with scores ranging from 0 to 13. The distribution of reporting quality across the two primary domains—completeness and clinical informativeness —revealed significant variation in data density. The completeness domain (VRQS 1–10) was led by high reporting for manufacturer (100.00%), vaccine type (97.92%), and demographics (sex: 87.61%, state: 83.55%). In comparison, critical traceability markers like lot numbers (66.14%) and dose series (62.74%) showed relatively lower rates. Data density was notably lower in the clinical informativeness domain (VRQS 11–13), where, despite 59.75% of reports featuring detailed clinical narratives, only 34.25% were professionally vetted, and only 16.18% contained informative laboratory evidence.

**Table 2:** Performance and distribution of the vaccine reporting quality score (VRQS) (N = 40,285).

VRQS item	Variable evaluated	Frequency (n)	Percentage (%)
Domain I. completeness			
VRQS1	Patient Age	31,292	77.68
VRQS2	Patient Sex	35,293	87.61
VRQS3	Reporting State	33,660	83.55
VRQS4	Plausible Onset Interval (0–365 days)	27,530	68.34
VRQS5	Report date present and ≥ vaccination date)	33,620	83.46
VRQS6	Administration Institution	26,553	65.91
VRQS7	Specific Vaccine Type	39,446	97.92
VRQS8	Specific Manufacturer	40,285	100.00
VRQS9	Vaccine Lot/Batch Number	26,645	66.14
VRQS10	Vaccine Dose Series	25,273	62.74
Domain II. clinical informativeness			
VRQS11	Professional Source	13,796	34.25
VRQS12	Informative Lab Data	6,519	16.18
VRQS13	Clinical Narrative (> 250 characters)	24,071	59.75
**Total Score**	Mean ± SD	9.04 ± 1.96	
**Quality Tier**	Score Range		
Low	0–7	8,092	20.09
Medium	8–10	23,181	57.54
High	11–13	9,012	22.37

Quality-tier score ranges are proposed purely as descriptive, exploratory groupings based on the baseline sample distribution; they are empirically unvalidated and do not correspond to an established clinical or psychometric diagnostic standard. Bold values indicate category-level summary statistics or domain headings; individual VRQS items are presented in plain text.

Based on the empirical distribution, reports were stratified into three quality tiers: low (0–7, 20.09%), medium (8–10, 57.54%), and high (11–13, 22.37%). To evaluate the robust nature of these intervals, validation checks were conducted across alternative data-driven grouping frameworks—including empirical tertiles, a median-based split, and quartile divisions (**Supplementary Material 2**, **Supplementary Table 3**). Due to the discrete integer nature of the 13-point score and a heavy concentration of reports at the peak scoring threshold (Median=10), purely mathematical cutoffs generated highly clumped and asymmetrical distribution buckets. Sensitivity checks utilizing empirical tertiles confirmed that our primary fixed quality tranches safely and logically preserve the gradient of vaccine safety reporting informativeness without introducing arbitrary cut-point distortions within tied indices.

### 3.2 Comparison of the VRQS by demographic and administrative factors

Analysis of VAERS reporting quality revealed significant variations across patient demographics and administrative contexts(p < 0.001 for all) (**Table 3**). Pediatric reports had the highest mean overall quality (9.90 ± 1.18), whereas reports for elderly patients had the lowest (9.68 ± 1.26). Female patient records had marginally higher overall quality (9.50 ± 1.53) than those of males (9.46 ± 1.54). Geographically, reports originating from the Territories scored the highest overall quality (9.97 ± 1.36), whereas the Northeast recorded the lowest (9.49 ± 1.51). Administratively, reports from public facilities yielded higher overall marks (10.03 ± 1.05) than private sites (9.90 ± 1.12). Professional reports exhibited substantially lower overall quality than spontaneous narratives (5.45 ± 2.18 vs. 9.22 ± 1.02; Cohen’s d = -1.40, 95% CI: -1.42, -1.38), mainly driven by lower completeness domain scores (5.45 ± 2.18 vs. 9.22 ± 1.02). In contrast, professional reports had higher average clinical informativeness (2.08 ± 0.28 vs. 0.59 ± 0.68). This subdomain divergence reflects an intrinsic structural characteristic of the data tracking system rather than behavioral variation, as professional designation is a direct component of the clinical informativeness metric.

**Table 3:** Comparison of VRQS total, completeness, and clinical informativeness by demographic and administrative characteristics (N = 40,285).

Characteristic	Category	n (%)	Completeness M ± SD	Clin. Info. M ± SD	Total VRQS M ± SD	Cohen’s d vs. ref (95% CI)	P
Age	Pediatric	9,421 (23.4)	9.05 ± 1.21	0.86 ± 0.85	9.90 ± 1.18	Ref	Ref
	Adult	12,614 (31.3)	8.96 ± 1.18	0.85 ± 0.82	9.81 ± 1.23	−0.08 (−0.11, −0.05)	<0.001
	Elderly	9,257 (23.0)	8.89 ± 1.33	0.80 ± 0.86	9.68 ± 1.26	−0.18 (−0.21, −0.15)	<0.001
	UNK	8,993 (22.3)	4.34 ± 1.66	2.03 ± 0.35	6.37 ± 1.69	−2.43 (−2.47, −2.39)	<0.001
Sex	Male	13,178 (32.7)	8.48 ± 1.78	0.98 ± 0.90	9.46 ± 1.54	Ref	Ref
	Female	22,115 (54.9)	8.53 ± 1.79	0.98 ± 0.89	9.50 ± 1.53	0.03 (0.01, 0.05)	0.011
	UNK	4,992 (12.4)	3.86 ± 1.71	1.99 ± 0.29	5.85 ± 1.69	−2.28 (−2.32, −2.24)	<0.001
Region	Northeast	5,631 (14.0)	8.42 ± 1.82	1.07 ± 0.90	9.49 ± 1.51	Ref	Ref
	Midwest	7,636 (19.0)	8.66 ± 1.68	0.94 ± 0.87	9.59 ± 1.44	0.07 (0.04, 0.11)	<0.001
	South	12,387 (30.7)	8.57 ± 1.74	1.00 ± 0.89	9.57 ± 1.47	0.05 (0.02, 0.09)	<0.001
	West	7,880 (19.6)	8.65 ± 1.71	0.98 ± 0.85	9.63 ± 1.46	0.10 (0.06, 0.13)	<0.001
	Territories	126 (0.3)	9.02 ± 1.47	0.94 ± 0.82	9.97 ± 1.36	0.32 (0.14, 0.50)	<0.001
	UNK	6,625 (16.4)	4.61 ± 2.23	1.66 ± 0.83	6.28 ± 1.82	−1.90 (−1.95, −1.86)	<0.001
Adm Site	Public	14,883 (36.9)	9.37 ± 0.86	0.66 ± 0.72	10.03 ± 1.05	Ref	Ref
	Private	9,660 (24.0)	9.27 ± 0.90	0.62 ± 0.72	9.90 ± 1.12	-0.12 (-0.15, -0.09)	<0.001
	Other	2,010 (5.0)	8.19 ± 1.76	1.21 ± 0.90	9.41 ± 1.63	-0.54 (-0.59, -0.50)	<0.001
	UNK	13,732 (34.1)	5.40 ± 2.10	1.90 ± 0.59	7.30 ± 2.05	-1.69 (-1.72, -1.67)	<0.001
Reporter	Spontaneous	26,489 (65.8)	9.22 ± 1.02	0.59 ± 0.68	9.82 ± 1.23	Ref	Ref
	Professional	13,796 (34.2)	5.45 ± 2.18	2.08 ± 0.28	7.53 ± 2.21	-1.40 (-1.42, -1.38)	<0.001

VRQS, Vaccine Reporting Quality Score; UNK, Unknown/Unspecified/Missing; Clin. Info., Clinical Informativeness. Some group comparisons (e.g., UNK age, UNK sex, UNK state, UNK admin site) are structurally related to the scoring system because age, sex, state, and admin site are VRQS items (1, 2, 3, 6). The clinical interpretation of these contrasts should account for the structural relationship.

### 3.3 Comparison of the VRQS by clinical history and case outcomes

Documenting pre-existing clinical risk histories was consistently associated with higher overall score quality (p < 0.001 for all comparisons; **Table 4**). Pre-existing patient allergies generated the most pronounced overall quality divergence (10.01 ± 1.10 vs. 8.77 ± 2.06; Cohen’s d = 0.66, 95% CI:0.63, 0.68), followed by prior adverse reactions (Cohen’s d = 0.45, 95% CI: 0.39, 0.52) and concurrent medications (Cohen’s d = 0.45, 95% CI: 0.43, 0.48). Patients with pre-existing clinical risk histories also showed a unique inverse relationship within sub-domains, yielding lower structural completeness but elevated narrative informativeness. Clinical severity and patient recovery similarly dictated documentation patterns (p < 0.001). SAEs exhibited lower structural completeness (7.86 ± 2.04 vs. 7.94 ± 2.37) but substantially higher clinical informativeness (1.38 ± 0.86 vs. 1.08 ± 0.91) and higher overall quality (9.24 ± 1.90 vs. 9.02 ± 1.96) than non-serious profiles. Lastly, reports for patients who fully recovered demonstrated higher overall quality than those who were not recovered (9.72 ± 1.53 vs. 9.36 ± 1.80; Cohen’s d = 0.21, 95% CI:0.19, 0.24).

**Table 4:** Comparison of VRQS total, completeness, and clinical informativeness by clinical history and case outcomes (N = 40,285).

Characteristic	Category	n (%)	Completeness M ± SD	Clin. Info. M ± SD	Total VRQS M ± SD	Cohen’s d vs. ref (95% CI)	P value
Clinical history							
Allergy	No	31,665 (78.6)	7.56 ± 2.48	1.21 ± 0.92	8.77 ± 2.06	Ref	Ref
	Yes	8,620 (21.4)	9.32 ± 0.86	0.69 ± 0.71	10.01 ± 1.10	0.66 (0.63, 0.68)	<0.001
Prior Reaction	No	39,240 (97.4)	7.90 ± 2.36	1.11 ± 0.91	9.01 ± 1.97	Ref	Ref
	Yes	1,045 (2.6)	9.12 ± 0.90	0.78 ± 0.73	9.90 ± 1.16	0.45 (0.39, 0.52)	<0.001
Current Illness	No	36,527 (90.7)	7.90 ± 2.38	1.09 ± 0.90	8.99 ± 1.98	Ref	Ref
	Yes	3,758 (9.3)	8.31 ± 1.96	1.18 ± 0.93	9.48 ± 1.65	0.25 (0.22, 0.29)	<0.001
Medical History	No	31,988 (79.4)	7.74 ± 2.44	1.14 ± 0.91	8.87 ± 2.01	Ref	Ref
	Yes	8,297 (20.6)	8.69 ± 1.77	0.96 ± 0.88	9.66 ± 1.60	0.40 (0.38, 0.43)	<0.001
Medication	No	31,547 (78.3)	7.73 ± 2.49	1.12 ± 0.92	8.85 ± 2.06	Ref	Ref
	Yes	8,738 (21.7)	8.68 ± 1.51	1.04 ± 0.88	9.72 ± 1.36	0.45 (0.43, 0.48)	<0.001
Case outcomes							
SAE	No	36,962 (91.8)	7.94 ± 2.37	1.08 ± 0.91	9.02 ± 1.96	Ref	Ref
	Yes	3,323 (8.2)	7.86 ± 2.04	1.38 ± 0.86	9.24 ± 1.90	0.11 (0.08, 0.15)	<0.001
Recovery	No	10,088 (25.0)	8.24 ± 2.09	1.12 ± 0.90	9.36 ± 1.80	Ref	Ref
	Yes	11,205 (27.8)	8.95 ± 1.74	0.77 ± 0.81	9.72 ± 1.53	0.21 (0.19, 0.24)	<0.001
	Unknown	18,992 (47.1)	7.17 ± 2.52	1.29 ± 0.91	8.46 ± 2.09	-0.45 (-0.47, -0.43)	<0.001

VRQS, Vaccine Reporting Quality Score; UNK, Unknown/Unspecified/Missing; Clin. Info., Clinical Informativeness. Bold text denotes major category headings and summary measures.

### 3.4 Multivariable regression analysis

Three regression models were estimated to disentangle the independent contributions of administrative, demographic, and clinical correlates associated with the total VRQS (Model 1), completeness domain (Model 2), and clinical informativeness domain (Model 3) (**Table 5**). To avoid compositional circularity, variables identified in the codebook as VRQS sub-score items (VRQS1–3, VRQS6, and VRQS11) were excluded *a priori* from the model in which their scores appear as dependent variables.

**Table 5:** Multivariable regression analysis of factors associated with VRQS total and domain scores (N = 40,285).

Variable	Category	Model 1: total VRQS (1-13)— OLS b (SE)	Model 2: completeness (1-10)— OLS b (SE)	Model 3: clin. info. (0-3)— ologit OR (95% CI)
Age Group	Pediatric	—	—	(Ref)
	Adult (18–64)	—	—	0.804 (0.758, 0.852)^***^
	Elderly (65+)	—	—	0.649 (0.609, 0.693)^***^
	Unknown	—	—	6.471 (5.822, 7.193)^***^
Gender	Male	—	—	(Ref)
	Female	—	—	1.070 (1.022, 1.119)^**^
	Unknown/Unidentified	—	—	1.058 (0.957, 1.170)
Region	Northeast	—	—	(Ref)
	Midwest	—	—	0.874 (0.815, 0.937)^***^
	South	—	—	0.920 (0.863, 0.981)^*^
	West	—	—	0.993 (0.927, 1.065)
	Territories	—	—	1.014 (0.719, 1.431)
	Unknown	—	—	0.599 (0.548, 0.654)^***^
Admin Site	Public	—	—	(Ref)
	Private	—	—	0.722 (0.683, 0.763)∗∗∗
	Other	—	—	2.517 (2.286, 2.772)∗∗∗
	Unknown	—	—	21.303 (19.694, 23.044)∗∗∗
Reporter	Spontaneous	—	(Ref)	—
	Professional	—	−3.648 (0.018)∗∗∗	—
Clinical History (No = Ref)	Allergies	0.832 (0.024)∗∗∗	0.084 (0.021)∗∗∗	0.831 (0.788, 0.876)^***^
	Prior Reaction	0.241 (0.058)∗∗∗	−0.196 (0.048)∗∗∗	1.106 (0.983, 1.244)
	Medication	0.463 (0.024)∗∗∗	0.386 (0.020)∗∗∗	1.302 (1.234, 1.373)^∗∗∗^
	Current Illness	0.011 (0.033)	0.156 (0.027)∗∗∗	1.527 (1.422, 1.641)^***^
	Medical History	0.116 (0.026)∗∗∗	−0.116 (0.021)∗∗∗	1.295 (1.224, 1.369)^***^
SAE	Yes (No = Ref)	−0.044 (0.034)	−0.563 (0.028)∗∗∗	2.746 (2.546, 2.961)^***^
Recovery	No	(Ref)	(Ref)	(Ref)
	Yes	0.403 (0.026)∗∗∗	0.204 (0.021)∗∗∗	0.684 (0.647, 0.723)^***^
	Unknown	−0.654 (0.024)∗∗∗	−0.116 (0.020)∗∗∗	0.595 (0.563, 0.629)^***^
Model Stats				
	R2/Pseudo-R2	0.1341	0.5936	0.2746
	Constant/Cutpoints	8.925 (0.022)∗∗∗	9.140 (0.019)∗∗∗	cut1 = -0.442; cut2 = 1.299; cut3 = 6.495

*Notes, Significance levels, *p < 0.05, **p < 0.01, ***p < 0.001. b represents the unstandardized coefficient. VRQS, Vaccine Reporting Quality Score; SAE, Serious Adverse Event; UNK, Unknown/Unspecified/Missing; Clin. Info., Clinical Informativeness. Model 1 (Total VRQS) and Model 2 (Completeness) are multivariate linear regression models, Model 3 (clinical inform) is an ordinal logistic regression. “—” = variable dropped from that model to avoid circularity. Model 1 (Total VRQS) drops age group, gender, region, admin site, and reporter (VRQS1-3, VRQS6, VRQS11). Model 2 (completeness) drops age group, gender, region, admin site (VRQS1-3, VRQS6). Model 3 (Clinical Informativeness) drops reporter (VRQS11). Bold text denotes major category headings and summary measures.

Model 1 (Total VRQS, OLS; *R*² = 0.1341) showed that documentation of allergies (b = 0.832, SE = 0.024, *p* < 0.001), concurrent medication (b = 0.463, SE = 0.024, *p* < 0.001), confirmed recovery (b = 0.403, SE = 0.026, *p* < 0.001), prior reaction (b = 0.241, SE = 0.058, *p* < 0.001), and medical history (b = 0.116, SE = 0.026, *p* < 0.001) were associated with higher report quality, while reports with unknown recovery status was associated with lower report quality (b = −0.654, SE = 0.024, *p* < 0.001). Current illness and SAE status showed no detectable association.

Model 2 (OLS; *R*² = 0.5936) revealed that professional reports were associated with substantially lower completeness scores than spontaneous reports (b = −3.648, SE = 0.018, *p* < 0.001). Positive associations were observed for medication (b = 0.386), confirmed recovery (b = 0.204), current illness (b = 0.156), and allergies (b = 0.084), each *with p < 0.001* and SEs of 0.020–0.027. Negative associations were observed for SAE status (b = −0.563, SE = 0.028), prior reaction (b = −0.196, SE = 0.048), medical history (b = −0.116, SE = 0.021), and unknown recovery (b = −0.116, SE = 0.020), all *p* < 0.001.

Model 3 (ordinal logistic, Pseudo-R² = 0.2746) demonstrated that adult (OR = 0.804, 95% CI 0.758–0.852) and elderly cohorts (OR = 0.649, 95% CI 0.609–0.693) had significantly lower odds of reaching higher clinical informativeness categories compared with pediatric records. Female reports had marginally higher odds than male reports (OR = 1.070, 95% CI 1.022–1.119). Relative to the Northeast, odds were lower in the Midwest (OR = 0.874) and South (OR = 0.920), but did not differ in the West, territories, or unknown regions. Private-administered reports had lower odds than public ones (OR = 0.722); the “other” (OR = 2.517) and “unknown” (OR = 21.303) admin-site categories showed much higher odds. Among clinical history variables, current illness (OR = 1.527), concurrent medication (OR = 1.302), and medical history (OR = 1.295) were positively associated, whereas allergies were negatively associated (OR = 0.831, 95% CI 0.788–0.876). SAEs had roughly threefold higher odds of higher informativeness (OR = 2.746, 95% CI 2.546–2.961). Compared with unrecovered patients, both recovered (OR = 0.684) and unknown-recovery (OR = 0.595) reports had lower odds of being clinically informative.

## 4 Discussion

### 4.1 Summary of the findings

This study proposes and applies a preliminary, exploratory 13-item scoring framework—the VRQS—designed to systematically summarize the quality of report documentation within the VAERS. Based on an evaluation of 40,285 adverse event records, our findings confirm that basic administrative data (such as vaccine type and manufacturer) are almost universally present, whereas essential clinical verification markers—such as informative laboratory data—are significantly less common. The empirical distribution suggests that documentation profiles vary significantly by report origin and clinical characteristics, rather than serving as a monolithic attribute of data quality.

A key finding is the apparent divergence between structural completeness (the systematic filling of administrative checkfields) and clinical informativeness (the depth of narrative and medical text). This performance is driven by opposing forces: Domain I (Completeness) is determined by the reporting pathway, with professional sources yielding lower administrative scores, while Domain II (Clinical Informativeness) is governed by clinical complexity, where SAEs, concurrent medications, and detailed medical histories significantly increase the probability of rich narrative and objective data. Rather than serving as a tool for causal validation, this proposed framework offers a transparent approach to prioritizing and screening reports before formal medical review. Consequently, quality-improvement initiatives must be tailored separately—focusing on administrative completeness within professional reporting channels and promoting clinical narrative depth across all submission types.

### 4.2 Evaluation of reporting quality

Application of the preliminary VRQS framework shows a mean of 9.04 ± 1.96, with 57.54% of reports clustering within a medium-quality tier. Similar to findings in global pharmacovigilance studies, such as those utilizing VigiGrade (9), basic demographic and product identifiers (e.g., manufacturer at 100.00%, vaccine type at 97.92%) are reported with high frequency. This aligns with literature suggesting that mandatory fields or easily accessible information are naturally prioritized in spontaneous reporting systems (13–15). In contrast, an administrative-to-clinical data imbalance is visible across the dataset. While nearly 60% of all submissions include a descriptive narrative exceeding 250 characters, informative laboratory data are exceptionally scarce (16.18%). This pattern highlights a distinct baseline characteristics gap in spontaneous reporting: reporters frequently supply detailed personal accounts of their experiences, yet the inclusion of objective medical testing remains low. This gap underscores that narrative text length does not inherently correlate with the density of objective clinical evidence required for downstream signal evaluation (16).

### 4.3 Documentation profiles by source and complexity

The data demonstrate a distinct structural divergence between routine clerical completeness and qualitative clinical informativeness across different reporting categories. Spontaneous reports submitted by the public yielded a high-completeness, low-informativeness profile. Previous studies on global databases have also noted that consumer reports frequently contain more complete information but lack the “technical informativeness” found in clinician-led reports (15, 17). Conversely, reports under the broad umbrella of professional sources exhibited an inverse footprint, displaying lower structural completeness but higher narrative and laboratory details. Crucially, grouping these sources as “professional” is a structural generalization that may obscure meaningful operational differences. Manufacturer reports, healthcare provider submissions, and public health program records differ substantially in their regulatory mandates, core objectives, and the original provenance of their data (18). Therefore, professional designation does not automatically guarantee superior clinical credibility or accuracy. The lower administrative completeness observed among these entries likely stems from time-constrained professional environments or localized database transmission formats that favor qualitative medical descriptions over checking routine administrative boxes (19).

A similar profile inversion was identified in relation to clinical severity and patient risk. Reports documenting SAEs or complex medical backgrounds (e.g., extensive allergy profiles or concurrent medication use) consistently showed lower structural completeness alongside higher clinical text density. In acute or high-stakes medical contexts, the documentation profile naturally shifts toward recording clinical progression, interventions, and diagnostic findings, which frequently leaves routine clerical fields unaddressed (20). However, caution must be exercised when interpreting these patterns. This divergence is not necessarily a reflection of intentional reporter motivations or behavioral priorities; rather, it represents a difference in documentation profiles driven by data-entry context and source characteristics.

### 4.4 Multivariable correlates of reporting quality

Multivariable regression models provide a quantitative look at the independent administrative and clinical correlates of report quality. However, interpreting these models requires addressing a major structural limitation: compositional circularity inherent in the regression design. Because specific baseline tracking variables (such as age, gender, geographic region, and reporting facility type) are embedded as direct scoring components within the completeness sub-score, evaluating their predictive impact on that same score introduces mathematical circularity. To minimize this bias, variables used as scoring criteria were excluded *a priori* from any model in which they were part of the dependent variable.

Accounting for these constraints, Model 1 demonstrates that overall documentation density is positively associated with underlying patient complexity, led by pre-existing allergies (b=0.832, p<0.001) and concurrent medications (b=0.463, p<0.001). Patients navigating complex clinical histories naturally generate a more extensive data trail. This complex history may assist reviewers in distinguishing background medical conditions from true vaccine-related events. The literature indicates that these comorbid factors often prompt reporters to be more thorough, helping investigators rule out alternative causes of the adverse event (21, 22). Within the individual domains, SAE status was associated with lower structural completeness (b=−0.563, p<0.001 in Model 2) but with nearly threefold higher odds of clinical informativeness (OR = 2.746, 95% CI: 2.546–2.961 in Model 3). Furthermore, confirmed patient recovery was positively associated with completeness (b=0.204, p<0.001), illustrating that resolved cases provide a static, easily documented timeline compared to actively evolving clinical courses. This follows the trend in the literature, where “closed” cases (those with a known outcome) provide a more complete narrative than “open” cases, in which the clinical course is still evolving at the time of report submission (23, 24). Together, these predictors underscore that high-quality reporting is driven by a combination of reporter expertise and the inherent clinical complexity of the patient’s history.

### 4.5 Practical applications

In practical application, the proposed VRQS is not designed to serve as an automated arbiter of clinical truth, nor is it intended to replace expert clinical evaluation; rather, its utility lies in preliminary database processing and exploratory research. Potential operational implementations include database screening and sensitivity analyses, where researchers can use the score to filter low-effort or fragmented records before executing signal-detection algorithms, or run parallel sensitivity analyses to determine whether a suspected signal holds across various documentation tiers. Additionally, the framework can facilitate report weighting and prioritization by helping safety teams identify complex, high-informativeness records for accelerated follow-up and clinical review. Finally, it can drive system improvements by pinpointing consistently omitted fields—such as vaccine lot numbers or dose sequences—to guide targeted updates to user interfaces and VAERS reporting forms, thereby encouraging more thorough data entry.

Importantly, a high VRQS score does not resolve the deep structural limitations inherent in passive surveillance. Broader systemic challenges—including widespread underreporting, media-driven stimulated reporting, notoriety bias, duplicate reporting, and the complete lack of a total population denominator—affect the interpretation of safety data even when individual reports achieve maximum quality scores. The VRQS measures documentation density and structural consistency within a report; it does not confirm the biological validity of an adverse event or verify clinical causality.

### 4.6 Limitations

The findings of this study must be interpreted in light of several key limitations. First, the VRQS is a proposed, unvalidated framework that has not been cross-validated against established global benchmarks, such as the WHO’s VigiGrade system. Its psychometric properties, consistency, and alignment with expert clinical consensus remain unproven, meaning all conclusions derived from its application must be treated as preliminary and exploratory until formal validation is completed. Second, this study relies on a cross-sectional dataset, offering only a single temporal snapshot of reporting profiles. Consequently, it does not capture how documentation quality shifts dynamically during public health emergencies, changing regulatory requirements, or the introduction of novel vaccine technologies. Third, because the dataset is restricted to passive surveillance, it is highly prone to selection bias that cannot be corrected by individual-report completeness metrics. Future research must focus on applying this proposed score to active surveillance systems and electronic health records to evaluate whether the observed documentation imbalances persist in controlled environments.

## 5 Conclusions

This study proposes and applies a preliminary 13-item scoring framework, the VRQS, to provide a transparent, exploratory approach for summarizing documentation quality within the VAERS database. Our analysis of 40,285 reports indicates that documentation profiles vary significantly across source categories and case severities. Spontaneous reports from the public exhibit high structural completeness but low clinical informativeness, whereas professional entries and serious adverse events systematically prioritize descriptive narrative and clinical context over administrative checkfields. Multivariable modeling confirms that reporting completeness is often lower in urgent clinical scenarios and highlights that missing administrative data fields often serve as structural artifacts of professional reporting styles. Crucially, administrative documentation density does not inherently correlate with clinical evidentiary value or patient recovery. Because the VRQS has not yet been externally validated, it cannot be considered a validated tool for causality assessment or automated signal detection. It is instead proposed as an exploratory screening tool to help prioritize records for detailed clinical review and to guide administrative improvements in safety reporting forms, pending formal validation against established quality measures.

## Data Availability

The data used in this study were obtained from the Vaccine Adverse Event Reporting System (VAERS), a publicly available database co-managed by the Centers for Disease Control and Prevention (CDC) and the Food and Drug Administration (FDA). The raw datasets can be accessed and downloaded directly from the VAERS website at https://vaers.hhs.gov/data.html. Data for the 2025 analysis were downloaded on April 1, 2026, and represent the reporting status as of that date. The specific datasets analyzed for this study (covering the 2025 reporting period) are available from the corresponding author upon reasonable request.
