# Supplementary material for: Development and application of the vaccine reporting quality score to evaluate the vaccine adverse event reporting system
Source: Front Immunol. 2026 Aug 6;17:1871141. doi: 10.3389/fimmu.2026.1871141 (PMC13490088; doi:10.3389/fimmu.2026.1871141)
Supplement: Supplementary file 1 [file DataSheet1.pdf]

Manuscript ID: 1871141 - Supplementary Material

Supplementary Material to: Development and Application of the Vaccine Reporting Quality Score (VRQS) to Evaluate the Vaccine Adverse Event Reporting System (VAERS).

Supplementary Material S1. VRQS Codebook

This codebook provides the exact rules used to construct the Vaccine Reporting Quality Score (VRQS) from the VAERS 2025 dataset (N = 40,285 unique VAERS_IDs after 1:1 merge of the patient-level file with the collapsed vaccine-level file). The codebook is intended to be used together with the deposited Stata do-file (Zenodo DOI: [FILL upon deposition]).

S1.1 Placeholder values

The following tokens are treated as placeholders (i.e., treated as missing for the purpose of item scoring). The list is applied to the four vaccine-level string fields (VAX_TYPE, VAX_MANU, VAX_LOT, VAX_DOSE_SERIES) and is implemented in the Stata do-file as a case-insensitive equality check after upper-casing and trimming the field:

- UNKNOWN
- UNK
- NONE
- NA
- N/A
- NOT PROVIDED
- "." (single period)
- "?" (single question mark)

A field is treated as non-missing if, after upper-casing and trimming whitespace, it is non-null in the VAERS data file AND not equal to any token in the placeholder list above.

S1.2 Multi-vaccine aggregation rule

Items 7, 8, 9, and 10 are vaccine-specific (i.e., their values are defined on the report-vaccine row dataset, not the report-level file). The vaccine dataset is first collapsed to one row per unique VAERS_ID using max() on the four binary indicators (has_vax_type, has_vax_manu, has_vax_lot, has_vax_dose) generated from the cleaned, placeholder-stripped values. The report is scored 1 on each item if the corresponding indicator is 1 for at least one vaccine row in the report; otherwise 0. The collapse and 1:1 merge are implemented in Steps 1-2 of the Stata do-file.

S1.3 Per-item operational definitions

All items are scored 1 = informative / plausible, 0 = missing or implausible, except VRQS1 (Age) and VRQS2 (Sex) which require a non-placeholder and (for Sex) non-Unknown value. Items 1-6 use the report-level (patient) file; items 7-10 use the collapsed vaccine-level file; item 11 uses the patient-level report-source field; item 12 uses the symptoms/lab-data field; item 13 uses the symptom narrative. Items 1-10 form the Completeness domain (range 0-10). Items 11-13 form

the Clinical Informativeness domain (range 0-3). The total VRQS is the row-sum of items 1-13 (range 0-13).

S1.3.1 VRQS1 - Age

Item	Field	Rule	Notes
**VRQS1 - Age**	AGE_YRS; CAGE_YR; CAGE_MO	AGE_YRS is non-missing. If AGE_YRS is missing, the item is filled from CAGE_YR (years, computed by VAERS) and, if still missing, from CAGE_MO (months) as a numeric value. If all three are missing, the item is scored 0 and the report is treated as UNK age.	The resulting continuous age (in years) is then used to construct the age-group variable AGEGROUP (1 = Pediatric <18; 2 = Adult 18-64; 3 = Elderly 65+; 4 = UNK).

S1.3.2 VRQS2 - Sex

Item	Field	Rule	Notes
**VRQS2 - Sex**	SEX	SEX is non-missing AND not equal to a placeholder AND not equal to "U". Valid informative values: F, M.	SEX is recoded into a numeric GENDER variable (1 = Male, 2 = Female, 3 = Missing/Unidentified). The UNK category is created when the value is missing, "U", or a placeholder.

S1.3.3 VRQS3 - State

Item	Field	Rule	Notes
**VRQS3 - State**	STATE	STATE is non-missing AND not equal to a placeholder. Valid informative values: 2-letter US state codes and US territory codes.	STATE is encoded into a numeric STATES variable. State is also mapped to a numeric REGION variable (1 = Northeast, 2 = Midwest, 3 = South, 4 = West, 5 = Territories) using a US Census Region classification.

S1.3.4 VRQS4 - Plausible onset interval

Item	Field	Rule	Notes
**VRQS4 - Plausible onset interval**	ONSET_DATE; VAX_DATE; RECVDATE	0 <= (ONSET_DATE - VAX_DATE) <= 365 days AND 0 <= (RECVDATE - ONSET_DATE) <= 365 days. Same-day onset and same-day report are scored 1.	Multi-vaccine handling: if the report contains multiple vaccines, the earliest VAX_DATE across vaccines in the report is used as VAX_DATE, and ONSET_DATE

		Records with missing ONSET_DATE, VAX_DATE, or RECVDATE, or with values outside the 0-365 range, are scored 0. The interval, report lag, and total time are computed as Stata elapsed-day numerics with %td formatting.	is treated as a single report-level value. The bounded 0-365 range is applied to both onset interval and report lag.

S1.3.5 VRQS5 - Timeliness (RECVDATE >= VAX_DATE)

Item	Field	Rule	Notes
**VRQS5 - Timeliness**	RECVDATE; VAX_DATE	RECVDATE is non-missing AND VAX_DATE is non-missing AND RECVDATE >= VAX_DATE.	This is a coarse administrative-timeliness marker. It is intended as a complement to VRQS4 (time-to-onset) and is not a substitute for time-to-report analyses.

S1.3.6 VRQS6 - Administration site

Item	Field	Rule	Notes
**VRQS6 - Administration site**	VAX_ADMIN_BY	VAX_ADMIN_BY is non-missing AND not equal to a placeholder. The encoded value is then mapped to a four-level ADMSITE_G (1 = public, 2 = private, 3 = other, 4 = unknown). ADMSITE_G = 4 (the VAX_ADMIN_BY code 9 "Unknown") is scored 0; all other non-missing codes are scored 1.	The VAX_ADMIN_BY categories PUB, PVT, OTH, MIL, WRK, SCH, UNK are preserved in the encoded numeric ADMSITE. The "Other" group aggregates MIL (military), WRK (workplace), SCH (school), and OTH.

S1.3.7 VRQS7 - Vaccine type

Item	Field	Rule	Notes
**VRQS7 - Vaccine type**	VAX_TYPE	VAX_TYPE is non-missing AND not equal to a placeholder token (after upper-casing and trimming). Multi-vaccine aggregation: report scored 1 if the criterion is satisfied for >=1 vaccine in the report.	Implemented as the has_vax_type binary indicator, generated at the vaccine-row level and collapsed (max) to the report level. Specific vaccine names (e.g., COVID19, FLU, HEP, MMR) are preserved for the vaccine-category distribution in Table 5.

S1.3.8 VRQS8 - Manufacturer

Item	Field	Rule	Notes
**VRQS8 - Manufacturer**	VAX_MANU	VAX_MANU is non-missing AND not equal to a placeholder token. Multi-vaccine aggregation: report scored 1 if the criterion is satisfied for >=1 vaccine in the report.	Implemented as the has_vax_manu binary indicator, generated at the vaccine-row level and collapsed (max) to the report level. Manufacturer names are preserved for downstream analysis (Table 5).

S1.3.9 VRQS9 - Lot number

Item	Field	Rule	Notes
**VRQS9 - Lot number**	VAX_LOT	VAX_LOT is non-missing AND not equal to a placeholder token. Multi-vaccine aggregation: report scored 1 if the criterion is satisfied for >=1 vaccine in the report.	Implemented as the has_vax_lot binary indicator, generated at the vaccine-row level and collapsed (max) to the report level. The lot number is described in the manuscript as an important traceability field.

S1.3.10 VRQS10 - Dose series

Item	Field	Rule	Notes
**VRQS10 - Dose series**	VAX_DOSE_SERIES	VAX_DOSE_SERIES is non-missing AND not equal to a placeholder token. Multi-vaccine aggregation: report scored 1 if the criterion is satisfied for >=1 vaccine in the report.	Implemented as the has_vax_dose binary indicator, generated at the vaccine-row level and collapsed (max) to the report level. Valid values include 1, 2, 3, 4, 5, 6, 7+, and special codes for boosters (e.g., BOOSTER).

S1.3.11 VRQS11 - Reporter (manufacturer / immunization project)

Item	Field	Rule	Notes
**VRQS11 - Reporter**	SPLTTYPE	SPLTTYPE is non-missing (i.e., the report is attributed to a manufacturer or to an immunization project, which are the VAERS reporter categories with a higher data-collection standard).	Implemented as the binary REPORTER indicator, value-labelled with ynlbl (1 = Yes, 0 = No). This item contributes to the Clinical Informativeness domain (range 0-3) in the primary analysis.

S1.3.12 VRQS12 - Informative laboratory data present

<table>
<tr><th>Item</th><th>Field</th><th>Rule</th><th>Notes</th></tr>
<tr><td>VRQS12 - Informative laboratory data</td><td>LAB_DATA</td><td>LAB_DATA is non-missing AND, after trimming whitespace, is non-empty AND does not match the garbage-pattern regular expression ^(none|no|n/a|na|unk|unknown|\.|\-|\?)$ (case-insensitive).</td><td>The garbage regex is applied to trimmed, lower-cased content. Records that contain a non-placeholder alphanumeric description are scored 1. The word "verified" is reserved for situations in which laboratory results have been confirmed by medical-record review or external validation; this study does not perform such verification.</td></tr>
</table>

S1.3.13 VRQS13 - Clinical narrative length

Item	Field	Rule	Notes
**VRQS13 - Clinical narrative length**	SYMPTOM_TEXT	Length of SYMPTOM_TEXT (in characters) > 250. Whitespace at the start and end is stripped before measuring length.	Sensitivity analyses at the 100, 500, and 1,000-character thresholds are also generated (VRQS13_100, VRQS13_500, VRQS13_1000) and reported in Supplementary Table S4. Spearman rank-order correlation of total VRQS across thresholds is 0.95-0.96.

S1.4 Domain and total scores

The thirteen items are aggregated into two domain scores and one total score using the Stata egen rowtotal() function:

- Completeness = VRQS1 + VRQS2 + VRQS3 + VRQS4 + VRQS5 + VRQS6 + VRQS7 + VRQS8 + VRQS9 + VRQS10. Range 0-10.
- Clinical Informativeness = VRQS11 + VRQS12 + VRQS13. Range 0-3.
- Total VRQS = sum of items 1-13. Range 0-13.

Three alternative total-score variants are also generated for sensitivity analysis: tVRQS_100, tVRQS_500, and tVRQS_1000, which replace VRQS13 with the corresponding 100-, 500-, and 1,000-character narrative-length indicator.

S1.5 Quality tiers

The primary quality-tier classification uses fixed cutoffs on the 0-13 total scale:

- Low (Quality = 1): tVRQS <= 7.
- Medium (Quality = 2): 8 <= tVRQS <= 10.

- High (Quality = 3): tVRQS >= 11.

Three data-driven alternative tiering strategies are generated and reported as sensitivity analyses:

- Empirical tertiles: xtile tier_tertile = tVRQS, nquantiles(3).
- Median-based binary split: xtile tier_median = tVRQS, nquantiles(2).
- Quartile-based four-group split: xtile tier_quartile = tVRQS, nquantiles(4).

Empirical centiles of tVRQS (25th, 33.3rd, 50th, 66.6th, 75th) are reported to support reader interpretation of the tier boundaries.

Supplementary Table S1. Mapping from VigiGrade dimensions to VRQS items

Each VigiGrade dimension is mapped to one of three fates in VRQS: retained, modified, or dropped, with the reason given.

VigiGrade dimension	VRQS item	Fate	Reason
Time to onset	VRQS4	Retained (modified)	Both measures the temporal relationship between exposure and event. VRQS adds an explicit 0–365-day plausibility window.
Indication	—	Dropped	VAERS does not include a structured indication field at the report level; indication is implicit in the vaccine type.
Outcome (incl. death)	SAE (covariate, not VRQS item)	Used as covariate	SAE is used as a covariate in the regression but is not a VRQS item, to avoid double-counting clinical events.
Sex	VRQS2	Retained	Direct field mapping (SEX).
Age	VRQS1	Retained	Direct field mapping (AGE_YRS).
Dose	VRQS10	Retained (modified)	VigiGrade uses a binary 'dose reported' indicator; VRQS uses the dose-series value (1, 2, 3, etc.).
Country / region	VRQS3	Retained	VAERS does not include country; VRQS uses

VigiGrade dimension	VRQS item	Fate	Reason
		(modified)	US state, aggregated to 5 Census regions.
Reporter type (health professional vs. other)	VRQS11	Retained (modified)	VRQS uses the VAERS CATEGORY field, which has 4 values; the dichotomous contrast used in the primary analysis aggregates the 3 professional categories.
Free-text (narrative length)	VRQS13	Retained (modified)	VigiGrade uses a binary 'narrative > X words' indicator; VRQS uses character length > 250 with sensitivity at 100/250/500/1,000.
Not in VigiGrade	VRQS7, VRQS8, VRQS9	Added by VRQS	Vaccine-specific traceability: vaccine type, manufacturer, and lot number are emphasized in VAERS but not in general ICSR tools.
Not in VigiGrade	VRQS5, VRQS12	Added by VRQS	Laboratory-data presence and an administrative-timeliness marker are added to capture clinical-informativeness dimensions relevant to vaccine reports.

Outcome. VigiGrade outcome is used as a covariate in the regression (SAE status) and is not a VRQS item. This avoids double-counting clinical events in the documentation-quality score.

Supplementary Table S2. Narrative-length threshold sensitivity (VRQS13)

Threshold (chars)	Reports ≥ threshold, n (%)	Mean total VRQS	Spearman ρ vs. primary	Notes
100	32,453 (80.56%)	9.24 ± 2.04	0.95***	Excludes short-length narratives.
250 (primary)	24,071 (59.75%)	9.04 ± 1.96	1.00***	Primary threshold; matches the original analysis.
500	17,983 (44.64%)	8.88 ± 1.87	0.96***	Excludes mid-length narratives.
1,000	12,053 (29.92%)	8.74 ± 1.88	0.95***	Restricts to long, detailed narratives.

VRQS13 is re-computed at four alternative thresholds (100, 250, 500, and 1,000 characters). The proportion of reports satisfying each threshold, the resulting mean total VRQS, and the Spearman rank-order correlation of the total VRQS across thresholds are reported. Across the four thresholds, the rank-order correlation of total VRQS is 0.95–0.96, indicating that the choice of threshold does not materially change the descriptive findings of the analysis.

*VRQS: Vaccine Reporting Quality Score. *** P<0.001*

Supplementary Table S3. Sensitivity Analysis of Alternative Categorical Grouping Cutoffs (N= 40, 285)

Grouping Strategy	Score Range	Number of Reports (n)	Proportion (%)	Methodological Justification & Notes
Original Cutoffs (Clinical / Fixed Intervals)				Primary Analysis Model. Establishes balanced, interpretable quality tiers independent of data clustering.
Low Quality	0−7	8,092	20.09	Baseline reference group.
Medium Quality	8−10	23,181	57.54	Moderate informativeness.
High Quality	11−13	9,012	22.37	Robust clinical and administrative profiles.
Strategy A: Empirical Tertiles (Data-Driven 3-Way Split)				Alternative Grouping. Generated dynamically via Stata's xtile command.
Low Tertile	≤8	19,102	47.42	Captures the lower half of the distribution.
Medium Tertile	9	12,171	30.21	Captures the center inflection point.
High Tertile	≥10	9,012	22.37	Matches the peak scoring cluster.
Strategy B: Median-Based Split (Data-Driven Binary Split)				Robustness Check. Evaluates the impact of shifting to a high vs. low binary classification threshold.
Below Median	≤9	31,273	77.63	Reflects the left-skewed nature of reporting patterns.
Above/At Median	≥10	9,012	22.37	The cutpoint is pulled upward due to high data density at score 10.
Strategy C: Quartile-Based Split (Data-Driven 4-Way Split)				Granular Check. Illustrates tie-breaking constraints caused by heavy data clumping.

Grouping Strategy	Score Range	Number of Reports (n)	Proportion (%)	Methodological Justification & Notes
1st Quartile (Lowest)	≤8	12,067	29.95	Lower-bound reporting tier.
2nd Quartile	9	19,206	47.68	Mid-tier concentration.
3rd Quartile	—	—	—	Omitted naturally; 50th to 75th percentiles share an identical score value of 10.
4th Quartile (Highest)	≥10	9,012	22.37	Top performing cluster.

Due to the discrete nature of the 13-point VRQS continuous score and high data density at the median value (Median=10), standard empirical median and quartile divisions resulted in heavily clumped, asymmetrical distribution buckets. Sensitivity checks utilizing data-driven empirical tertiles confirmed that our primary categorical hardcoded tranches (0-7 Low, 8-10 Medium, 11-13 High) safely and logically preserve the gradient of vaccine safety reporting informativeness without introducing arbitrary cut-point distortions within tied indices. The proportion of reports in each tier is sensitive to the choice of cutoffs, but the overall pattern of associations is unchanged. We recommend that future versions of VRQS report the total score primarily as a continuous variable.

Supplementary Figure S1.

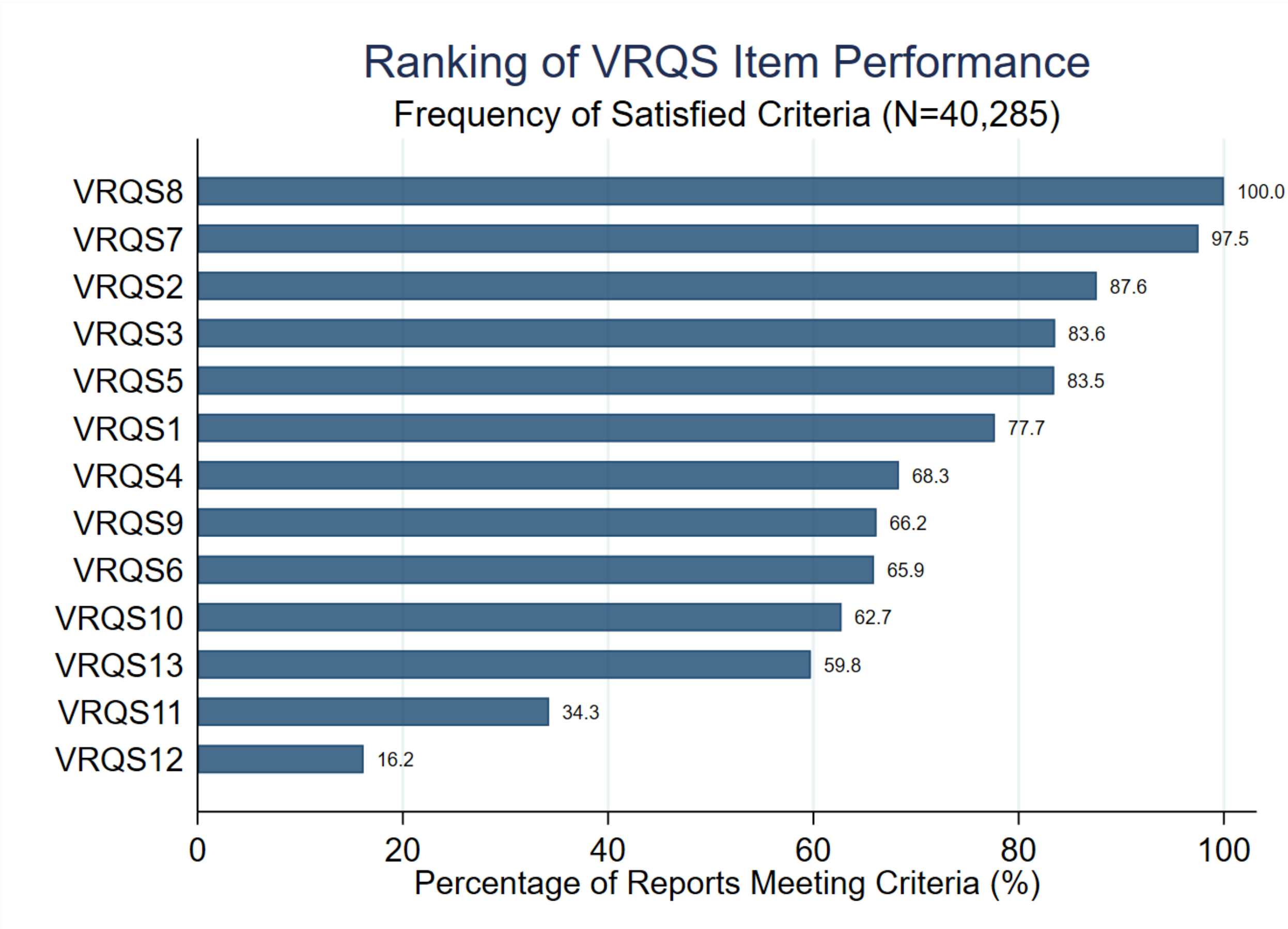

Figure S1. Comparative Performance Across VRQS Criteria

Figure Legend: This horizontal bar chart illustrates the frequency of satisfied criteria for each Vaccine Reporting Quality Score (VRQS) item across 40, 285 analyzed reports. Individual components are ranked from highest to lowest frequency, with the x-axis representing the percentage of reports meeting each specific criterion
