# Supplementary material for: Development and application of the vaccine reporting quality score to evaluate the vaccine adverse event reporting system
Source: Front Immunol. 2026 Aug 6;17:1871141. doi: 10.3389/fimmu.2026.1871141 (PMC13490088; doi:10.3389/fimmu.2026.1871141)
Supplement: Supplementary file 2 [file DataSheet2.pdf]

```
**********************************************************
***Data merge  Unique vaers_id (n=40285)
**********************************************************


*
=============================================================================================
=====
* STEP 1: LOAD, CLEAN, AND COLLAPSE THE VACCINE DATASET (202502.dta)
*
=============================================================================================
=====
use "C:\4. Paper 2026\00. VEARS\3. Quality index\202502.dta", clear
keep vaers_id vax_type vax_manu vax_lot vax_dose_series

* Define common non-informative placeholders used in VAERS text entry
local placeholders ` " "UNKNOWN" "UNK" "NONE" "NA" "N/A" "NOT PROVIDED" "." "?" "'

* Clean strings and strip out placeholders for the four key target fields
foreach var in vax_type vax_manu vax_lot vax_dose_series {
   capture confirm string variable `var'
   if !_rc {
      quietly replace `var' = upper(trim(`var'))
      foreach p in `placeholders' {
         quietly replace `var' = "" if `var' == "`p'"
      }
```

```
   }
}

* Generate binary indicator variables tracking if valid data exists on a given row
gen byte has_vax_type = (!missing(vax_type) & vax_type != "")
gen byte has_vax_manu = (!missing(vax_manu) & vax_manu != "")
gen byte has_vax_lot  = (!missing(vax_lot)  & vax_lot != "")
gen byte has_vax_dose = (!missing(vax_dose_series) & vax_dose_series != "")


* Collapse multi-vaccine records to a single unique row per report
collapse (max) has_vax_type has_vax_manu has_vax_lot has_vax_dose, by(vaers_id)

* Save this as a physical temporary file in your directory
save "C:\4. Paper 2026\00. VEARS\3. Quality index\temp_collapsed_vax.dta", replace

*
======================================================================
=====
* STEP 2: LOAD PATIENT DATA AND EXECUTE CLEAN 1:1 MERGE
*
======================================================================
=====
use "C:\4. Paper 2026\00. VEARS\3. Quality index\202501.dta", clear

* Deduplicate the patient file first (as you did in your code snippet)
bysort vaers_id (onset_date): keep if _n == 1
```

* Perform the 1:1 merge using the absolute path to the file we just saved

merge 1:1 vaers_id using "C:\4. Paper 2026\00. VEARS\3. Quality index\temp_collapsed_vax.dta"

*
==
=====

* STEP 3: VERIFICATION AND CLEAN UP

*
==
=====

tab _merge

drop if _merge == 2

drop _merge

* Erase the temporary file from your hard drive so it stays clean

erase "C:\4. Paper 2026\00. VEARS\3. Quality index\temp_collapsed_vax.dta"

* Check final analytical unit count

count

****

***Data cleaning

****

```
*****************
***Demographics
******************
**age
replace age_yrs= cage_yr + cage_mo if age_yrs==.
rename age_yrs age

gen agegroup = .
replace agegroup = 1 if age < 18
replace agegroup = 2 if age >= 18 & age < 65
replace agegroup = 3 if age >= 65 & !missing(age)
replace agegroup = 4 if missing(age)
label define age_lab 1 "Pediatric (<18)" 2 "Adult (18-64)" 3 "Elderly (65+)" 4"unknown"
label values agegroup age_lab
label variable agegroup "Patient Age Category"

*** Create the binary variable VRQS1 (0=missing, 1=nonmissing)
gen VRQS1 = !missing(age)
label var VRQS1 "VRQS Item 1: Age is non-missing (1=Yes, 0=No)"


**gender
gen sex_num = .
replace sex_num = 1 if sex == "M"
replace sex_num = 2 if sex == "F"
replace sex_num = 3 if sex == "U" | sex == ""
```

```
label define sex_lab 1 "Male" 2 "Female" 3 "Missing/Unidentified"

label values sex_num sex_lab

label variable sex_num "Biological Sex (Recoded)"

rename sex_num gender


*** Create the binary variable VRQS2 (0=missing, 1=nonmissing)

gen VRQS2 = !missing(sex) & sex != "U"

label var VRQS2 "VRQS Item 2: Sex is non-missing (1=Yes, 0=No)"


**state

encode state, gen(states)

label variable states "U.S. State/Territory (Numeric)"


*** Create the binary variable VRQS3 (0=missing, 1=nonmissing)

gen VRQS3 = !missing(states)

label var VRQS3 "VRQS Item 3: States is non-missing (1=Yes, 0=No)"


***region

* Create a new numeric variable

gen region = .

* 1. Northeast

replace region = 1 if inlist(state, "CT", "ME", "MA", "NH", "RI", "VT", "NJ", "NY", "PA")

* 2. Midwest (Breaking into multiple lines to avoid r(130))

replace region = 2 if inlist(state, "IL", "IN", "MI", "OH", "WI")

replace region = 2 if inlist(state, "IA", "KS", "MN", "MO", "NE")

replace region = 2 if inlist(state, "ND", "SD")
```

```
* 3. South (Breaking into multiple lines to avoid r(130))
replace region = 3 if inlist(state, "DE", "DC", "FL", "GA", "MD", "NC", "SC")
replace region = 3 if inlist(state, "VA", "WV", "AL", "KY", "MS", "TN", "AR")
replace region = 3 if inlist(state, "LA", "OK", "TX")

* 4. West
replace region = 4 if inlist(state, "AZ", "CO", "ID", "MT", "NV")
replace region = 4 if inlist(state, "NM", "UT", "WY", "AK", "CA")
replace region = 4 if inlist(state, "HI", "OR", "WA")
* 5. Territories (Optional)
replace region = 5 if inlist(state, "AS", "GU", "MP", "PR", "VI")
replace region =6 if region==.
label define reg_lab 1 "Northeast" 2 "Midwest" 3 "South" 4 "West" 5 "Territories" 6
"Missing/Unidentified"
label values region reg_lab
label variable region "U.S. Census Region"

**Dates
gen recv_num = date(recvdate, "MDY")
gen vax_num = date(vax_date, "MDY")
gen onset_num = date(onset_date, "MDY")

format recv_num vax_num onset_num %td

gen interval=onset_num- vax_num
gen report_lag = recv_num - onset_num
```

```
gen total_time = recv_num - vax_num

*** Create the binary variable VRQS4 (0=missing, 1=nonmissing)

gen VRQS4 = 1
replace VRQS4 = 0 if numdays==.|numdays > 365 | numdays < 0|report_lag > 365|
report_lag  < 0
label var VRQS4 "VRQS Item 4: onset interval is non-missing and plausible (1=Yes, 0=No)"

** Construct VRQS5 (timeliness): RECVDATE >= VAX_DATE
gen VRQS5 = 0
replace VRQS5 = 1 if !missing(recv_num) & !missing(vax_num) & recv_num >= vax_num
label var VRQS5 "VRQS Item 5: report date present and >= vaccination date"

***administration site
encode v_adminby, gen(admsite)
label variable admsite "offacility administering the vaccine"

gen admsite_g=1 if admsite==2|admsite==4 |admsite==5|admsite==7|admsite==10
replace admsite_g=2 if admsite==1|admsite==6 |admsite==8
replace admsite_g=3 if admsite==3
replace admsite_g=4 if admsite==9
label define admglbl 1 "public" 2"private" 3"other" 4"unknow"
```

```
label values admsite_g admglbl

*** Create the binary variable VRQS6 (0=missing, 1=nonmissing)

gen VRQS6 = 1

replace VRQS6 = 0 if admsite==9

label var VRQS6 "VRQS Item 6: administration site is non-missing (1=Yes, 0=No)"



***vaccine type

rename has_vax_type VRQS7

label var VRQS7 "VRQS Item 7: Specific Vaccine Type provided (1 pt)"


**vaccine manufacture

rename has_vax_manu VRQS8

label var VRQS8 "VRQS Item 8: Specific Vaccine manufacture provided (1 pt)"


**vaccine lot

 rename has_vax_lot VRQS9

 label var VRQS9 "VRQS Item 9: Specific Vaccine lot provided (1 pt)"


 **vaccine dose

  rename has_vax_dose VRQS10

   label var VRQS10 "VRQS Item 10: Specific Vaccine dose provided (1 pt)"


***reporter

gen reporter =1 if !missing(splttype)

replace reporter =0 if reporter ==.
```

label var reporter "whether reported by a manufacturer or immunization project (1=Yes, 0=No)"

label values reporter ynlbl

*** Create the binary variable VRQS11 (0=missing, 1=nonmissing)???

gen VRQS11 = 0 if reporter==0

replace VRQS11 =1 if VRQS11==.

label var VRQS11 "VRQS Item 11: reported by a manufacturer or immunization project with a higher standard of data collection (1=Yes, 0=No)"

***Diagnostic Lab Data

*** Diagnostic Lab Data Evaluation (VRQS Item 12)

* 1. Define a strict list of strings that explicitly represent completely uninformative placeholders

* This captures exact matches for none, no, n/a, na, unk, unknown, dashes, periods, or question marks.

local garbage "^(?i)(none|no|n/a|na|unk|unknown|\.|\-|\?)$"

* Initialize your target score variable to 0

gen byte VRQS12 = 0

* 2. Execute the evaluation conditional logic using clean string quotes

```
* We trim the data first and check if it contains actual descriptions that do NOT match the garbage pattern

replace VRQS12 = 1 if !missing(lab_data) & trim(lab_data) != ""
& !ustrregexm(trim(lab_data), "`garbage'")


* 3. Apply standard metadata labels

label var VRQS12 "VRQS Item 12: Informative Lab Data (1 pts)"


* 4. Quality Control Check: Review the records scored as 0 to ensure accuracy

list lab_data if VRQS12 == 0 & !missing(lab_data) in 1/20



**** VRQS13: Narrative Granularity (Symptom Text) & Sensitivity analysis---

* Assign 1 points if the narrative is detailed (>250 characters)

* We use the strlen() function to count characters

gen VRQS13 = 0

replace VRQS13 = 1 if strlen(symptom_text) > 250 & !missing(symptom_text)

label var VRQS13 "VRQS Item 13: Narrative Length >250 chars (1 pts)"


gen VRQS13_100 = 0

replace VRQS13_100 = 1 if strlen(symptom_text) > 100 & !missing(symptom_text)

label var VRQS13_100 "VRQS Item 13_100: Narrative Length >100 chars (1 pts)"


gen VRQS13_500 = 0

replace VRQS13_500 = 1 if strlen(symptom_text) > 500 & !missing(symptom_text)

label var VRQS13_500 "VRQS Item 13_500: Narrative Length >500 chars (1 pts)"
```

```
gen VRQS13_1000 = 0

replace VRQS13_1000 = 1 if strlen(symptom_text) > 1000 & !missing(symptom_text)

label var VRQS13_1000 "VRQS Item 13_1000: Narrative Length >1000 chars (1 pts)"


* Generate the total score (Range: 0 - 13)

egen tVRQS = rowtotal(VRQS1 VRQS2 VRQS3 VRQS4 VRQS5 VRQS6 VRQS7 VRQS8 VRQS9
VRQS10 VRQS11 VRQS12 VRQS13)


egen tVRQS_100 = rowtotal(VRQS1 VRQS2 VRQS3 VRQS4 VRQS5 VRQS6 VRQS7 VRQS8
VRQS9 VRQS10 VRQS11 VRQS12 VRQS13_100)


egen tVRQS_500 = rowtotal(VRQS1 VRQS2 VRQS3 VRQS4 VRQS5 VRQS6 VRQS7 VRQS8
VRQS9 VRQS10 VRQS11 VRQS12 VRQS13_500)


egen tVRQS_1000 = rowtotal(VRQS1 VRQS2 VRQS3 VRQS4 VRQS5 VRQS6 VRQS7 VRQS8
VRQS9 VRQS10 VRQS11 VRQS12 VRQS13_1000)



tab1 VRQS13 VRQS13_100 VRQS13_500 VRQS13_1000

sum tVRQS tVRQS_100 tVRQS_500  tVRQS_1000

* Generate a complete Spearman correlation matrix for all sensitivity thresholds

spearman tVRQS tVRQS_100 tVRQS_500  tVRQS_1000, stats(rho p) bonferroni



* ---------------------------------------------------------------------------

* Total and domain scores on the 0-13 scale

* ---------------------------------------------------------------------------
```

```
* Completeness = sum(items 1-10), range 0-10

egen completeness = rowtotal(VRQS1 VRQS2 VRQS3 VRQS4 VRQS5 VRQS6 VRQS7 VRQS8
VRQS9 VRQS10)

label var completeness "Completeness domain (0-9)"


* Clinical Informativeness = sum(items 11-13), range 0-3

egen clininf = rowtotal(VRQS11 VRQS12 VRQS13)

label var clininf "Clinical Informativeness domain (0-4)"


* Check correlation between breadth (completeness) and depth (Clinical Informativeness)

pwcorr completeness clininf, sig


* Create Categories

gen quality = 1 if tVRQS <= 7

replace quality = 2 if tVRQS>= 8 & tVRQS <= 10

replace quality = 3 if tVRQS >= 11


label define qlabel 1 "Low (0-7)" 2 "Med (8-10)" 3 "High (11-13)"

label values quality qlabel

tab quality

*
=============================================================================
=====

* SENSITIVITY ANALYSIS: ALTERNATIVE TIER CUTOFFS BASED ON DATA DISTRIBUTION
Supplementraty Table S3

*
=============================================================================
=====
```

```
* ---------------------------------------------------------------------------
* 1. STRATEGY A: EMPIRICAL TERTILES (Primary Strategy - 3 Groups)
* ---------------------------------------------------------------------------
xtile tier_tertile = tVRQS, nquantiles(3)

label define lbl_tert 1 "Low Tertile" 2 "Medium Tertile" 3 "High Tertile"
label values tier_tertile lbl_tert
label var tier_tertile "Sensitivity Tier: Empirical Tertiles"

display "=== DISTRIBUTION FOR STRATEGY A: EMPIRICAL TERTILES ==="
tabulate tier_tertile



* ---------------------------------------------------------------------------
* 2. STRATEGY B: MEDIAN-BASED SPLIT (2 Groups)
* ---------------------------------------------------------------------------
xtile tier_median = tVRQS, nquantiles(2)

label define lbl_med 1 "Below Median" 2 "Above/At Median"
label values tier_median lbl_med
label var tier_median "Sensitivity Tier: Median-Based Split"

display "=== DISTRIBUTION FOR STRATEGY B: MEDIAN-BASED SPLIT ==="
tabulate tier_median
```

```
* -----------------------------------------------------------------------------

* 3. STRATEGY C: QUARTILE-BASED SPLIT (4 Groups)

* -----------------------------------------------------------------------------

xtile tier_quartile = tVRQS, nquantiles(4)


label define lbl_quart 1 "1st Quartile (Lowest)" 2 "2nd Quartile" 3 "3rd Quartile" 4 "4th
Quartile (Highest)"

label values tier_quartile lbl_quart

label var tier_quartile "Sensitivity Tier: Quartile-Based Split"


display "=== DISTRIBUTION FOR STRATEGY C: QUARTILE-BASED SPLIT ==="

tabulate tier_quartile



*
==================================================================================
=====

* OPTIONAL: SHOW ACTUAL EMPIRICAL CUTOFF SCORES FOR YOUR PAPER

*
==================================================================================
=====

centile tVRQS, centile(25 33.3 50 66.6 75)


******************

********Patient outcomes

******************
```

```
***clinic visit

gen docvisit = 0

replace docvisit = 1 if ofc_visit == "Y"

label values docvisit ynlbl

label variable docvisit "Healthcare Provider Office Visit"



***death

gen death = 0

replace death = 1 if died == "Y"

label define ynlbl 1 "Yes" 0 "No"

label values death ynlbl

label variable death "Death Reported (Recoded)"


***lifethreatening

gen threat = 0

replace threat= 1 if l_threat == "Y"

label values threat ynlbl

label variable threat "Life-Threatening Event Reported"


**Emergency Room

gen er = 0

replace er = 1 if er_visit == "Y" |er_ed_visit== "Y"

label values er ynlbl

label variable er "Unified Emergency Room/Dept Visit"
```

```
* Hospitalization/Prolonged Hospitalization
gen hospt = 0
replace hospt = 1 if hospital == "Y"
label values hospt ynlbl
label variable hospt "Hospitalization Reported"

gen lstay = 0
replace lstay = 1 if x_stay  == "Y"
label values lstay ynlbl
label variable lstay "Prolonged Hospital Stay Reported"

replace hospt=1 if lstay==1

* Permanent Disability
gen disability = 0
replace disability  = 1 if disable == "Y"
label values disability  ynlbl
label variable disability "Permanent Disability Reported"

* Birth Defect
gen birthdefect = 0
replace birthdefect = 1 if birth_defect == "Y"
label values birthdefect ynlbl
label variable birthdefect "Birth Defect Reported"

* recovery status
```

```
gen recovery = .

replace recovery = 1 if recovd== "Y"

replace recovery = 0 if recovd == "N"

replace recovery = 2 if recovd == "U" | recovd == ""


label define rec_lab 1 "Recovered" 0 "Not Recovered" 2 "Unknown/Missing"

label values recovery rec_lab

label variable recovery "Patient Recovery Status"


* Serious Adverse Event (SAE)

gen sae = 0

replace sae = 1 if death == 1 | threat == 1 | hospt == 1| lstay == 1|  disability==1|
birthdefect==1

label define sae_lab 1 "Serious" 0 "Non-Serious"

label values sae sae_lab

label variable sae "Serious Adverse Event Composite"


*******************************

******Clinical vulnerability

*********************************

***Concurrent Medications

gen othermeds = 1

replace othermeds = 0 if missing(other_meds)

local keywords "unk unknon none unsure not n.a n/a unknown no nothing"


foreach k in `keywords' {
```

```
   replace othermeds = 0 if strpos(strlower(other_meds), "`k'") > 0
}

label define meds_cat 0 "None/Unknown/Negative" 1 "Concurrent Meds Reported"
label values othermeds meds_cat
label variable othermeds "Patient Concurrent Medications (Refined)"



***concurrent Illness at Time of Vaccination
gen disease = 1
replace disease = 0 if missing(cur_ill)
local keywords "unk unknon none unsure not n.a n/a unknown no nothing"

foreach k in `keywords' {
   replace disease = 0 if strpos(strlower(cur_ill), "`k'") > 0
}

label define disease_cat 0 "None/Unknown/Negative" 1 "Current Illness Reported"
label values disease disease_cat
label variable disease "Acute Illness at Time of Vaccination"

* Pre-existing Medical History
gen medhistory = 1
replace medhistory = 0 if missing(history)
local keywords "unk unknon none unsure not n.a n/a unknown no nothing"
```

```
foreach k in `keywords' {
   replace medhistory = 0 if strpos(strlower(history), "`k'") > 0
}

label define hist_cat 0 "None/Unknown/Negative" 1 "Medical History Reported"
label values medhistory hist_cat
label variable medhistory "Pre-existing Medical History (Refined)"

***Prior Adverse Vaccine Reaction
gen vaxhistory = 1
replace vaxhistory = 0 if missing(prior_vax)
local keywords "unk unknon none unsure not n.a n/a unknown no nothing"

foreach k in `keywords' {
   replace vaxhistory = 0 if strpos(strlower(prior_vax), "`k'") > 0
}

label define vaxhist_cat 0 "None/Unknown/Negative" 1 "Prior Reaction Reported"
label values vaxhistory vaxhist_cat
label variable vaxhistory "Prior Adverse Vaccine Reaction Reported"

***Pre-existing Patient Allergies
gen allergy = 1
replace allergy = 0 if missing(allergies)
local keywords "unk unknon none unsure not n.a n/a unknown no nothing"
```

```
foreach k in ` keywords' {
   replace allergy = 0 if strpos(strlower(allergies), "` k'") > 0
}

label define allergy_cat 0 "None/Unknown/Negative" 1 "Allergies Reported"
label values allergy allergy_cat
label variable allergy "Pre-existing Patient Allergies"


***********************
Table 1 & 2: Principles and Overview of the VRQS System
**************************
tab1 VRQS*  quality
sum tVRQS  completeness clininf

tab1 gender agegroup region admsite_g  reporter  othermeds disease medhistory
vaxhistory allergy sae recovery, m


************************
Table 3 & 4 : Comparison of VRQS score by case characteristics
**************************

* 1. Check baseline record sample size to verify the analytical frame
count
display "Total dataset sample size must equal 40,285."
```

```
* Define macro lists for the three dependent score variables

local outcomes "tVRQS completeness clininf"


* ------------------------------------------------------------------------------

* SECTION I: SAMPLE DISTRIBUTION (n, %)

* ------------------------------------------------------------------------------

* Generate the counts and percentages for each categorical dimension in your layout

foreach var in agegroup gender region allergy vaxhistory disease medhistory othermeds
reporter sae admsite_g recovery {

   display "=== DISTRIBUTION FOR CHARACTERISTIC: `var' ==="

   tabulate `var'

}


*
================================================================================
=====

* SECTION II: BIVARIABLE OUTCOME PROFILE BY CHARACTERISTICS (SAFE UNROLLED
VERSION)

*
================================================================================
=====


* ------------------------------------------------------------------------------

* A. MULTI-CATEGORY PREDICTORS (Age Group, Gender, Region, Admin Site, Recovery)

* ------------------------------------------------------------------------------

foreach var in agegroup gender region admsite_g recovery {
```

```
    display
"=============================================================================
=="
    display " ANALYSIS FOR MULTI-CATEGORY VARIABLE: `var'"
    display
"=============================================================================
=="

    * 1. Outcome: tVRQS
    display "--> Mean +/- SD and ANOVA for tVRQS:"
    tabulate `var', summarize(tVRQS)
    oneway tVRQS `var'

    * 2. Outcome: completeness
    display "--> Mean +/- SD and ANOVA for completeness:"
    tabulate `var', summarize(completeness)
    oneway completeness `var'

    * 3. Outcome: clininf
    display "--> Mean +/- SD and ANOVA for clininf:"
    tabulate `var', summarize(clininf)
    oneway clininf `var'
}


* ---------------------------------------------------------------------------
* B. BINARY PREDICTORS (Allergies, Reaction History, Illness, Meds, Reporter, SAE)
```

```
* ---------------------------------------------------------------------------

foreach var in allergy vaxhistory disease medhistory othermeds reporter sae {

  display
"=============================================================================
=="

  display " ANALYSIS FOR BINARY LOGICAL PREDICTOR: `var'"

  display
"=============================================================================
=="


  * 1. Outcome: tVRQS

  display "--> Mean +/- SD, t-test, and Cohen's d for tVRQS:"

  tabulate `var', summarize(tVRQS)

  ttest tVRQS, by(`var')

  esize twosample tVRQS, by(`var') cohen


  * 2. Outcome: completeness

  display "--> Mean +/- SD, t-test, and Cohen's d for completeness:"

  tabulate `var', summarize(completeness)

  ttest completeness, by(`var')

  esize twosample completeness, by(`var') cohen


  * 3. Outcome: clininf

  display "--> Mean +/- SD, t-test, and Cohen's d for clininf:"

  tabulate `var', summarize(clininf)

  ttest clininf, by(`var')

  esize twosample clininf, by(`var') cohen
```

```
}

*************
Table 3. regression
*************
* Model 1: OLS of tVRQS
*   Drop: agegroup gender region admsite_g reporter (VRAS1-3, VRAS6, VRAS11)
regress tVRQS ///
   i.allergy i.vaxhistory i.disease i.medhistory ///
   i.othermeds i.sae i.recovery

* Model 2: OLS of completeness
*   Drop: agegroup (VRAS1), gender (VRAS2), region (VRAS3), admsite_g (VRAS6)
regress completeness ///
   i.reporter i.allergy i.vaxhistory i.disease ///
   i.medhistory i.othermeds i.sae i.recovery

* Model 3: ologit of clininf
*   Drop: reporter (VRAS11)
ologit clininf ///
   i.agegroup i.gender i.region i.admsite_g ///
   i.allergy i.vaxhistory i.disease i.medhistory ///
   i.othermeds i.sae i.recovery, or

************
```

```
**Figure S1
*************
* 1. Input data
clear
input str6 varname percent
"VRQS8" 100.00
"VRQS7" 97.52
"VRQS2" 87.61
"VRQS3" 83.55
"VRQS5" 83.46
"VRQS1" 77.68
"VRQS4" 68.34
"VRQS9" 66.15
"VRQS6" 65.91
"VRQS10" 62.74
"VRQS13" 59.75
"VRQS11" 34.25
"VRQS12" 16.18
end

* 2. Generate the ranked horizontal bar chart
graph hbar (asis) percent, ///
   over(varname, sort(1) descending) ///
   blabel(bar, format(%3.1f) size(vsmall)) ///
   ytitle("Percentage of Reports Meeting Criteria (%)") ///
   title("Ranking of VRQS Item Performance") ///
```

```
subtitle("Frequency of Satisfied Criteria (N=40,285)") ///

bar(1, color(navy%80)) ///

ylabel(0(20)100) ///

graphregion(color(white))

        i.othermeds i.disease i.medhistory i.vaxhistory ///

        i.allergy i.sae i.recovery, beta
```
